# Collective Intelligence Meets Medical Decision-Making: The Collective Outperforms the Best Radiologist

**DOI:** 10.1371/journal.pone.0134269

**Published:** 2015-08-12

**Authors:** Max Wolf, Jens Krause, Patricia A. Carney, Andy Bogart, Ralf H. J. M. Kurvers

**Affiliations:** 1 Leibniz Institute of Freshwater Ecology and Inland Fisheries, Müggelseedamm 310, 12587, Berlin, Germany; 2 Departments of Family Medicine and Pubic Health & Preventive Medicine, Knight Cancer Institute, Oregon Health & Science University, 3181 S.W. Sam Jackson Park Road, Portland, Oregon, United States of America; 3 RAND Corporation, 1776 Main Street, Santa Monica, CA, 90407–2138, United States of America; 4 Faculty of Life Sciences, Humboldt-University of Berlin, Berlin, Germany; University of Tuebingen Medical School, GERMANY

## Abstract

While collective intelligence (CI) is a powerful approach to increase decision accuracy, few attempts have been made to unlock its potential in medical decision-making. Here we investigated the performance of three well-known collective intelligence rules (“majority”, “quorum”, and “weighted quorum”) when applied to mammography screening. For any particular mammogram, these rules aggregate the independent assessments of multiple radiologists into a single decision (recall the patient for additional workup or not). We found that, compared to single radiologists, any of these CI-rules both increases true positives (i.e., recalls of patients with cancer) and decreases false positives (i.e., recalls of patients without cancer), thereby overcoming one of the fundamental limitations to decision accuracy that individual radiologists face. Importantly, we find that all CI-rules systematically outperform even the best-performing individual radiologist in the respective group. Our findings demonstrate that CI can be employed to improve mammography screening; similarly, CI may have the potential to improve medical decision-making in a much wider range of contexts, including many areas of diagnostic imaging and, more generally, diagnostic decisions that are based on the subjective interpretation of evidence.

## Background

Beliefs in individual experts and genius are deeply engrained in western societies yet research on collective intelligence has shown that groups can often outperform individuals when solving cognitive problems [1–4]. One of the earliest example is provided by Galton [5], who showed that the weight of an ox can be determined almost perfectly by pooling a large number of individual guesses. Over the last decades, impressive feats of collective intelligence (CI) have been described in a wide range of animal species including microbes, insects, fish, birds and humans [6–12]. In the human domain, a key challenge exists in identifying those contexts where decisions can be improved with CI, and to design decision-making systems that unlock this potential [13–15].

To date, relatively few studies have applied CI to the field of medical decision-making (but see [16–18]). Here we investigated the scope for CI among radiologists independently interpreting mammograms. Breast cancer is the most prevalent cancer type in females and currently accounts for 29% of all new cancer cases in the U.S. with relatively consistent incidence rates since 2003 [19]. Mammography is the prime diagnostic tool for early detection of breast cancer and also the most commonly used radiological screening method. During interpretation of screening mammograms, physicians face a trade-off between achieving a high true positive rate (i.e., the proportion of cancer cases correctly recalled for additional workup, or sensitivity) and a low false positive rate (i.e., the proportion of non-cancer cases incorrectly recalled for additional workup, or 1—specificity) [20,21]. Interpretations by a single radiologist as done in the U.S. and independent double reading of mammograms by two radiologists in combination with consensus discussion in cases of discordant opinions as done in Europe are the most common evaluation methods [22]. We stress that, despite substantial improvements in mammography screening, considerable scope for CI remains. According to the current Mammography Factsheet of the National Cancer Institute (U.S. Department of Health and Human Services), for example,”screening mammograms miss about 20 percent of breast cancers that are present at the time of screening” [23].

## Materials and Methods

In order to assess the potential for CI in mammography screening, we investigated the performance of three well-known CI-rules (Table 1). For any given mammogram, these rules integrate the independent assessments of multiple radiologists into a final decision (i.e., recall the patient for additional workup or not). The three rules differ in how they aggregate the individual assessments (‘recall’ or ‘no recall’) into a final decision and how much prior knowledge is required for their implementation (Table 1). Specifically, these rules prescribe that a patient is recalled whenever (i) a majority of the independent individual assessments is ‘recall’ (‘majority’), (ii) the frequency of independent individual assessments for ‘recall’ is higher than a pre-established quorum threshold (‘quorum’) and (iii) the frequency of the weighted independent individual assessments for ‘recall’ is higher than a pre-established quorum threshold (‘weighted quorum’). Importantly, all three CI-rules are predicted to increase the decision accuracy of groups compared to single decision makers [24–26].

**Table 1:** Three CI-rules^1^.

	Decision rule	Promotes collective intelligence whenever	Information requirement
**Majority**	Recall patient whenever a majority of the assessments is ‘recall’.	Each individual decision maker has an accuracy above 50%.	None.
**Quorum**	Recall patient whenever the fraction of the ‘recall’ assessments is higher than the pre-established quorum threshold.	The quorum threshold is set between the average true and false positive rate of decision makers.	The average true and false positive rate of decision makers.
**Weighted quorum**	As ‘quorum’, but the votes of individual decision makers are weighted according to their individual performance.	As ‘quorum’.	The accuracy of individual decision makers.

^1^ Note that these CI-rules are a sequence of increasingly complex rules: the majority rule is a special case of the quorum rule with the quorum threshold set to 0.5, and the quorum rule is a special case of the weighted quorum rule with the individual weights set to 1.0.

To investigate the performance of these CI-rules, we used one of the largest available mammography datasets, consisting of 16,813 interpretations by 101 radiologists of 182 mammograms in a test set study setting [27,28]. All cases included in the test set were randomly selected from screening examinations performed on women aged 40 to 69 between 2000 and 2003 from six U.S. mammography registries participating in the Breast Cancer Surveillance Consortium (S1 Text). Each screening examination included both current and previous views for comparison, consisting of craniocaudal (CC) and mediolateral oblique (MLO) views of each breast (4 views per woman for each of the screening and comparison examinations), which is standard practice in the U.S. [27]. As this dataset contains the independent assessments by multiple radiologists of the same mammogram (mean number of independent readings per mammogram = 92), and the true status of each mammogram (S1 Text), it allows us to investigate the performance of the above CI-rules. We stress that, while the above dataset has recently been used to investigate the performance of individual radiologists [29–31], up to now, its potential to investigate CI in mammography screening has not yet been harnessed.

Throughout, we composed groups of size *n* (range: 1 to 15) by randomly drawing *n* radiologists from the total pool of radiologists. To assess the performance of the majority rule (Table 1) and how this depends on group size we determined, for each mammogram, whether the majority of the *n* radiologists classified the mammogram as ‘recall’ or ‘no recall’. We only used odd group sizes to avoid the need for a tie-breaker rule. After classifying all mammograms in this way (i.e. following the majority), we used the known cancer status of each mammogram to calculate the average true and false positive rate and the overall accuracy (i.e., the proportion of mammograms which are correctly classified) achieved when employing this procedure. To assess the performance of groups that employ the quorum rule (Table 1) we randomly assigned half of the mammograms to a training set and the other half to a validation set. The training set was used to calibrate the quorum threshold which, in order to achieve gains in both true and false positives, has to be set below the average true positive rate and above the average false positive rate of the individual radiologists [26]. We thus calculated the average true and false positive rate of the *n* radiologists in the training set and set the quorum threshold halfway between these values (see below for alternative ways of setting the quorum threshold). We then evaluated the performance of the quorum rule in the validation set. For each mammogram in the validation set we determined the fraction of the *n* radiologists that classified the mammogram as ‘recall’. If this fraction was higher than or equal to the quorum threshold, then the mammogram was classified as ‘recall’, if not as ‘no recall’. After classifying all mammograms in the validation set, we used the known cancer status of each mammogram to calculate the average true and false positive rate and the overall accuracy of the quorum rule. Our weighted quorum rule (Table 1) resembles the quorum rule but additionally prescribes that the assessments of each radiologist is weighted according to her previous performance (S1 Text). In particular, we employ a commonly employed heuristic decision rule for binary choice scenarios as investigated by us [24,32,33]: if the performance of radiologist *i* is *p*_*i*_, then its vote has to be weighted by $w_{i}\;=\;\text{log}(\frac{p_{i}}{1-p_{i}})$. We thus followed the same procedure as described for the quorum rule, but used the training set to estimate the performance of each radiologist and used these estimates to set the weights in the validation set (S1 Text). For each CI-rule and each group size *n*, we repeated this procedure 2,500 times (including new and independent assignments of the mammograms to the training set and to the validation set) and then calculated the mean (± SEM). We compared this to the average performance and the performance of the best radiologist in each group (S1 Text).

## Ethics Statement

Data used for this research were collected during the course of a mammographic test set study designed to evaluate an intervention designed to improve mammographic accuracy among community radiologists [27,31]. The study involved radiologists from U.S. breast cancer registries including Carolina Mammography Registry, San Francisco Mammography Registry, New Hampshire Mammography Network, New Mexico Mammography Project, Vermont Breast Cancer Surveillance System, and Group Health Cooperative in western Washington, all affiliated with the Breast Cancer Surveillance Consortium (BCSC). The data were assembled at the BCSC Statistical Coordinating Center (SCC) in Seattle and analysed at the Leibniz-Institute of Freshwater Ecology and Inland Fisheries (IGB) in Berlin, Germany. All registries as well as the SCC and IGB received institutional review board approval for either active or passive consenting processes or a waiver of consent to enroll participants, pool data and perform statistical analysis. All procedures are in accordance with the Health Insurance Portability and Accountability Act and all data were anonymized to protect the identities of women, radiologists and facilities.

## Results and Discussion

**Fig 1:**
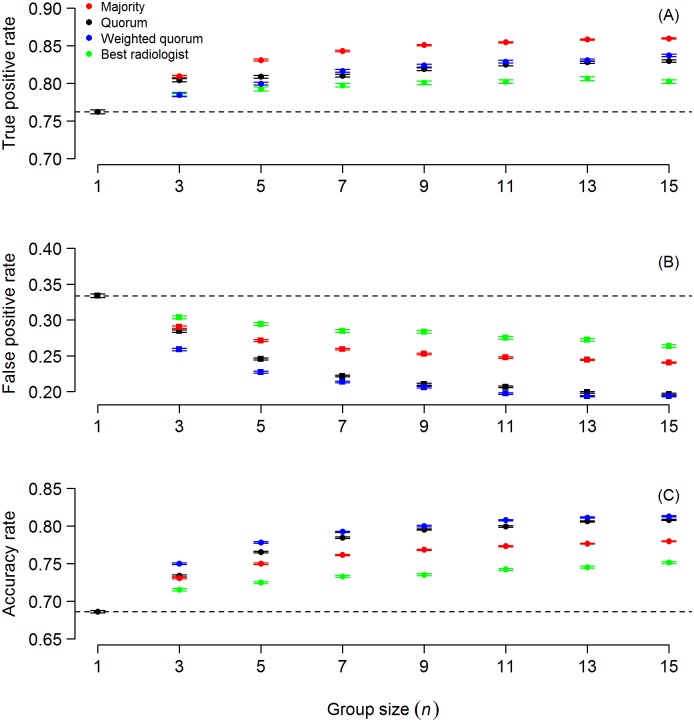
All three CI-rules outperform the best radiologist. All three CI-rules overcome the trade-off between true and false positives that single radiologists face, and outperform the best radiologist within each group. Shown are mean (± SEM) true positives (A), false positives (B) and accuracy (C) of the three CI-rules, as a function of group size *n*. The dashed line corresponds to the average individual performance of radiologists (i.e., group size of 1), the green dots correspond to the highest-performing radiologist for a given group size *n*.

Fig 1 shows the results from these analyses. We find that, as group size increases, all three CI-rules achieve both increases in true positives (Fig 1A) and decreases in false positives (Fig 1B). As a consequence, larger groups make more accurate decisions (Fig 1C). The simultaneous improvements in true and false positives is remarkable, as the trade-off between true and false positives is one of the fundamental constraints of decision accuracy that single radiologists face [20,21]. Our results show that each of the three CI-rules alleviates this constraint. Interestingly, gains achieved from larger group sizes level off around a group size of nine, after which adding more radiologists only has a marginal effect (Fig 1A to 1C). We stress that even relatively small group sizes can achieve substantial performance improvements (Fig 1). As expected, the performance of the highest-performing radiologist (green dots in Fig 1A to 1C) increases with increasing group size. This is because larger groups have a higher likelihood of harbouring high performers. Despite this, we find that groups employing any of the CI rules outperform the best-performing radiologist in that group, achieving more true positives (Fig 1A), fewer false positives (Fig 1B) and thus higher overall accuracy (Fig 1C).

When comparing the overall decision accuracy of the different rules, we find that the weighted quorum rule slightly outperforms the quorum rule and that the quorum rule outperforms the majority rule (Fig 1C). This was to be expected, as these three rules can be seen as a series of increasingly complex rules with the feature that the less complex rules are a special case of the more complex rules: the quorum rule results in the majority rule when the quorum threshold is set to 0.5, the weighted quorum rule results in the quorum rule when the individual weights are set to 1. The same performance ranking can be found for the false positives (Fig 1B). The fact that the majority rule achieves a higher true positive rate than either quorum rule (Fig 1A) can be explained by the particular way we set the quorum in the above analyses (i.e. halfway between the average true and false positive rate of radiologists), which favours performance gains in false positives (see below).

**Fig 2:**
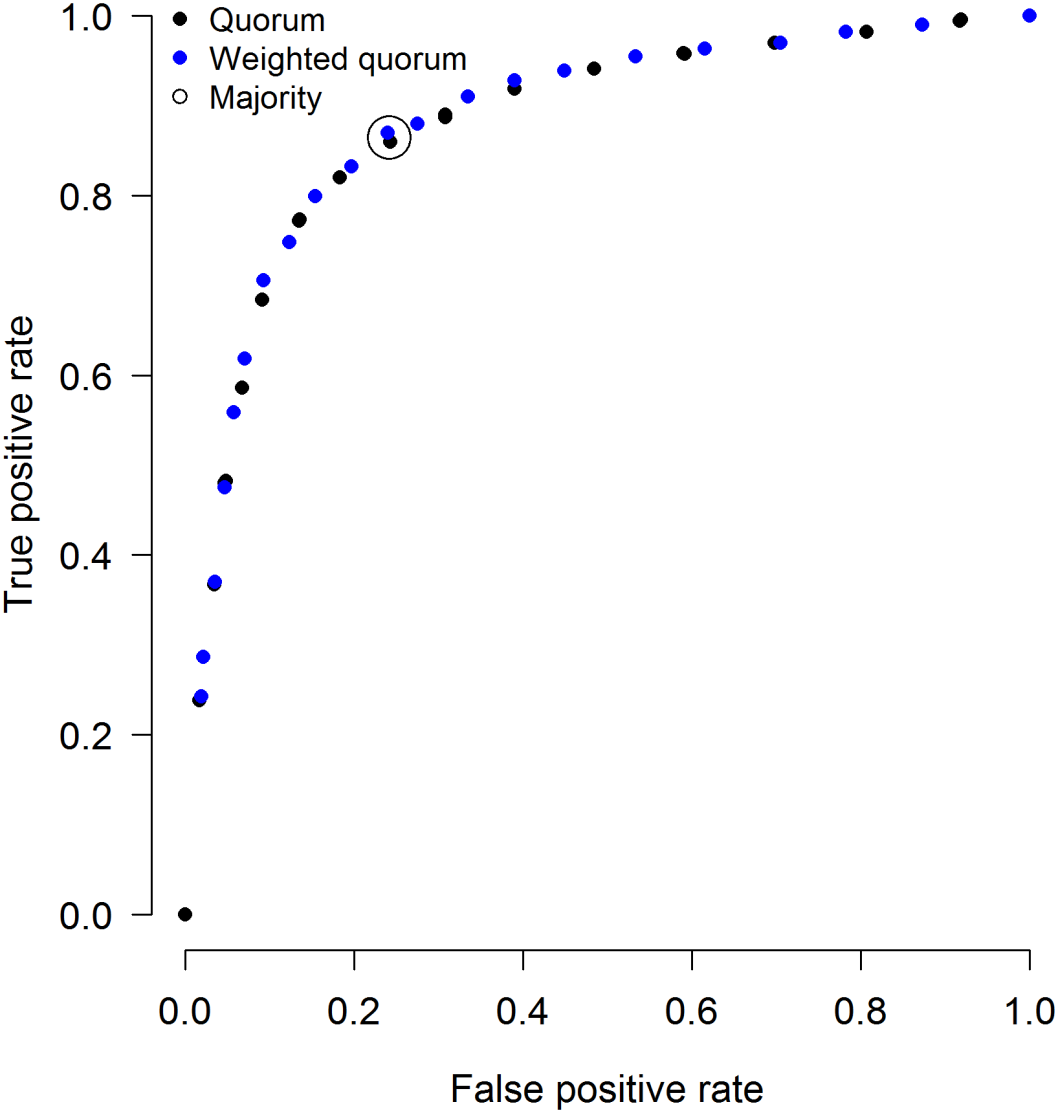
ROC curve for the quorum rule and the weighted quorum rule. Each dot is obtained by setting a different fixed quorum threshold, starting at 0 with increments of 0.05 up to 1. Data are based on a group size of 15 and 2,500 simulations (see main text). The majority rule corresponds to a fixed quorum threshold of 0.5. Note that, while we here consider the consequences of fixed quorum thresholds, the analyses in Fig 1 and Fig 3 are based on flexible quorum thresholds that are estimated from a training set (see main text).

The majority rule is the simplest and most parsimonious of the three rules, as its implementation does not require any information about the performance of decision makers. In order to implement the quorum rules, estimates of either the average true and false positive rate of decision makers (‘quorum’) or the individual performances (‘weighted quorum’) are needed. However, the quorum rules are more broadly applicable and more flexible than the majority rule. The majority rule is predicted to promote CI [24,25] only when individual decision makers have a decision accuracy above 50% (as is the case in the present data set; average true positive rate = 0.762, average false positive rate = 0.336). The quorum rules are not constrained by this condition and are predicted to promote CI whenever the quorum is set between the average true and false positive rates of the individuals involved in the decision [26,34]. Moreover, in contrast to the majority rule, the quorum rules can be fine-tuned in order to put more weight on gains in either true positives, false positives or overall accuracy. This fine-tuning is achieved by adjusting the quorum threshold: lower thresholds will increase true positive rate at the cost of lower gains in false positives, as fewer radiologists are required to assess a mammogram as ‘recall’ in order to recall a patient. Analogously, higher thresholds improve (i.e. reduce) the false positive rate at the cost of lower gains in true positives. This basic dependency of the true and false positive rates on the quorum threshold is illustrated in Fig 2, which shows the true and false positive rates of groups of size 15 employing one out of a broad range of fixed quorum thresholds (range: 0 to 1, with increments of 0.05), illustrating the trade-off between the true and false positive rate at the collective level.

**Fig 3:**
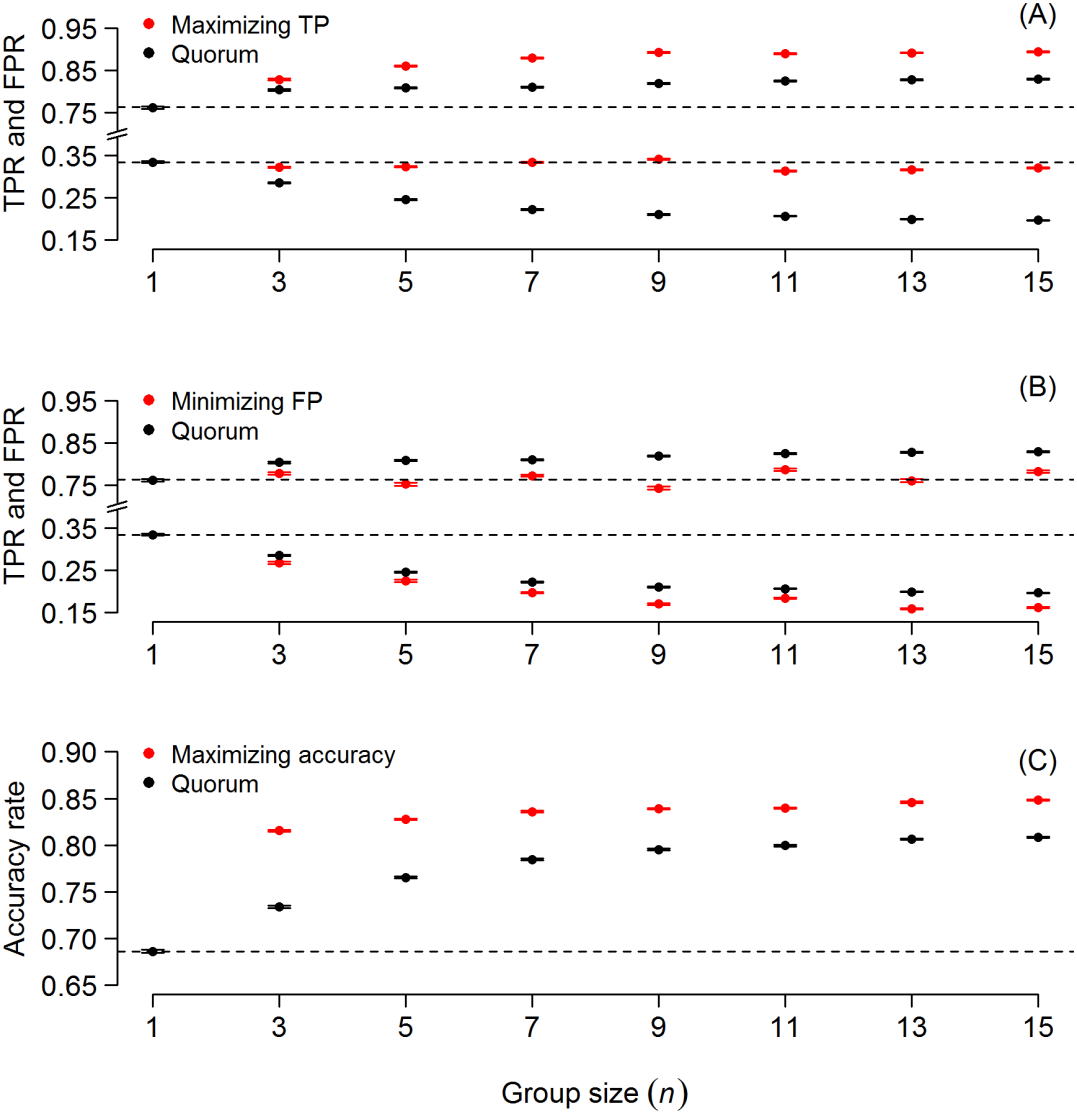
The quorum rule can be fine-tuned to put more weight on improving true positives, false positives or overall accuracy. The three panels correspond to the three illustrative scenarios where the goal was to: (A) maximize gains in true positives while keeping the false positive rate (FPR) close to the average false positive rate of 0.336 in the data set; (B) minimize the false positive rate while keeping the true positive rate (TPR) at the average true positive rate of 0.762 in the data set; and (C) maximize overall accuracy. As can be seen, on top of the gains achieved by a quorum threshold set halfway between the average true and false positive rate (black dots, corresponding to the values from Fig 1), further improvements in true positives (A), false positives (B) or overall accuracy (C) can be achieved by fine-tuning the quorum threshold appropriately. Shown are mean (± SEM).

To further illustrate the flexibility of the quorum rules, we considered three different scenarios where the goal is to: (i) maximize gains in true positives while keeping the false positive rate close to the average false positive rate of 0.336 in the data set (Fig 3A); (ii) minimize the false positive rate while keeping the true positive rate close to the average true positive rate of 0.762 in the data set (Fig 3B); and (iii) maximize overall accuracy (Fig 3C). For each scenario, we randomly selected groups of *n* radiologists (range: 1 to 15, odd numbers only) and used half of the mammograms as a training set and half as a validation set. In the training set we determined the quorum thresholds which resulted in (i) a false positive rate of 0.336, (ii) a true positive rate of 0.762 and (iii) maximum overall accuracy (S1 Text). We then investigated the performance of these thresholds in the validation set. Repeating this procedure 2,500 times (for each of the three scenarios, and each group size *n*) we find that, on top of the gains achieved by a quorum threshold set halfway between the average true and false positive rate (as in Fig 1), further improvements in true positives (Fig 3A), false positives (Fig 3B) or overall accuracy (Fig 3C) can be achieved by fine-tuning the quorum threshold appropriately. For example, while single radiologists on average achieve a true positive rate of 0.762, a group of five radiologists can achieve a true positive rate of 0.861 with a similar false positive rate as single individuals (Fig 3A). Alternatively, while single radiologists on average achieve a false positive rate of 0.336, a group of five radiologists can achieve a false positive rate of 0.226 with a similar true positive rate as single individuals (Fig 3B).

A common practice in the U.S. is to employ single interpretation of mammograms in combination with computer-aided detection (CAD). Compared to single reading without CAD, this practice generally increases true positives while also increasing false positives [35,36]. In contrast, our findings suggest that any of the three CI-rules can increase true positives and decrease false positives simultaneously. A common practice in Europe is independent double reading of mammograms in combination with consensus discussion in cases of discordant opinions. Generally, this practice achieves a higher true positive rates than single reading, and either increases or decreases the false positive rates depending on the recall policy [37–40]. While our data set does not allow us to directly compare the CI-rules to such consensus decisions, we stress that the proposed CI-rules have two key advantages. First, the dynamics of consensus discussions are inherently complex, and prone to well-known performance-reducing effects like group think [41,42]. In contrast, the above CI-rules provide highly transparent and algorithmic collective decision rules. Moreover, they exclude direct interactions between radiologists, thereby avoiding the negative consequences of group think and maintaining opinion diversity, a well-known condition for CI [1–4]. Second, consensus decisions often require that specialists meet and discuss, whereas the above mechanisms only requires independent assessments, thereby saving valuable time.

## Conclusion

Our findings suggest that simple and highly transparent CI-rules can be employed to improve the accuracy of mammography screening. Most likely, at least two factors contribute to the observed CI-effect. First, whenever errors (i.e. false positives and false negatives) are relatively rare and not perfectly correlated between radiologists, the CI-rules can effectively filter out these errors. Second, radiologists typically differ in their experience or ability with particular types of cases and the CI-rules can exploit this diversity. Of course, viewing time of specialists is costly and has to be taken into account. In fact, a substantial proportion of mammograms may be unambiguous and may thus not require more than two independent assessments. In such cases, one may envisage a decision tree in which a mammogram first gets assessed independently by two radiologists, and only in cases of disagreements is it evaluated by using the above CI-rules.

While we have focused here on mammography screening, our findings suggest that CI may have the potential to improve medical decision-making in a much wider range of contexts, including many areas of diagnostic imaging and, more generally, diagnostic decisions that are based on the subjective interpretation of evidence. Intriguingly, next to improving accuracy, CI may also pave the way to shared medical decision-making, thereby alleviating doctors of the sole responsibility for single cases.

## Supporting Information

S1 Text Data collection and data analysis. (DOCX)

## References

[1] Bonabeau E, Dorigo M, Theraulaz G (1999) Swarm Intelligence: From Natural to Artificial Systems. Oxford: Oxford University Press.

[2] Surowiecki J (2005) The wisdom of crowds: Random House LLC.

[3] Couzin ID (2009) Collective cognition in animal groups. Trends in Cognitive Sciences 13: 36–43. 10.1016/j.tics.2008.10.002 19058992

[4] Krause J, Ruxton GD, Krause S (2010) Swarm intelligence in animals and humans. Trends in Ecology and Evolution 25: 28–34. 10.1016/j.tree.2009.06.016 19735961

[5] Galton F (1907) Vox populi. Nature 75: 450–451.

[6] Franks NR, Pratt SC, Mallon EB, Britton NF, Sumpter DJ (2002) Information flow, opinion polling and collective intelligence in house–hunting social insects. Philosophical Transactions of the Royal Society of London Series B: Biological Sciences 357: 1567–1583. 12495514 10.1098/rstb.2002.1066 PMC1693068

[7] Arrow KJ, Forsythe R, Gorham M, Hahn R, Hanson R, Ledyard JO, et al (2008) Economics—The promise of prediction markets. Science 320: 877–878. 10.1126/science.1157679 18487176

[8] Woolley AW, Chabris CF, Pentland A, Hashmi N, Malone TW (2010) Evidence for a Collective Intelligence Factor in the Performance of Human Groups. Science 330: 686–688. 10.1126/science.1193147 20929725

[9] Ward AJW, Herbert-Read JE, Sumpter DJT, Krause J (2011) Fast and accurate decisions through collective vigilance in fish shoals. Proceedings of the National Academy of Sciences of the United States of America 108: 2312–2315. 10.1073/pnas.1007102108 21262802 PMC3038776

[10] Eckstein MP, Das K, Pham BT, Peterson MF, Abbey CK, Sy JL, et al (2012) Neural decoding of collective wisdom with multi-brain computing. Neuroimage 59: 94–108. 10.1016/j.neuroimage.2011.07.009 21782959

[11] Berdahl A, Torney CJ, Ioannou CC, Faria JJ, Couzin ID (2013) Emergent Sensing of Complex Environments by Mobile Animal Groups. Science 339: 574–576. 10.1126/science.1225883 23372013

[12] Ross-Gillespie A, Kümmerli R (2014) Collective decision-making in microbes. Frontiers in microbiology 5. 10.3389/fmicb.2014.00054 PMC3939447 24624121

[13] Sorkin RD, Hays CJ, West R (2001) Signal-detection analysis of group decision making. Psychological Review 108: 183–203. 11212627 10.1037/0033-295x.108.1.183

[14] Bahrami B, Olsen K, Latham PE, Roepstorff A, Rees G, Frith CD (2010) Optimally Interacting Minds. Science 329: 1081–1085. 10.1126/science.1185718 20798320 PMC3371582

[15] Barr S, Gold JM (2014) Redundant Visual Information Enhances Group Decisions. Journal of Experimental Psychology-Human Perception and Performance 40: 2124–2130. 10.1037/a0038224 25365569

[16] Ferrell WR, Hillmann BJ, Brewer ML, A mendola MA, Thornbury JR (1989) Interactive, mathematical, and sequential consultative methods in diagnosing renal masses on excretory urograms. Investigative Radiology 24: 456–462. 2521127 10.1097/00004424-198906000-00008

[17] Metz CE, Shen J-H (1992) Gains in accuracy from replicated readings of diagnostic images prediction and assessment in terms of ROC analysis. Medical Decision Making 12: 60–75. 1538634 10.1177/0272989X9201200110

[18] Landman BA, Asman AJ, Scoggins AG, Bogovic JA, Stein JA, Prince JL (2012) Foibles, follies, and fusion: Web-based collaboration for medical image labeling. NeuroImage 59: 530–539. 10.1016/j.neuroimage.2011.07.085 21839181 PMC3195954

[19] Siegel R, Ma J, Zou Z, Jemal A (2014) Cancer statistics, 2014. CA: A Cancer Journal for Clinicians 64: 9–29. 24399786 10.3322/caac.21208

[20] Metz CE (1978) Basic principles of ROC analysis. Seminars in Nuclear Medicine 8: 283–298. 112681 10.1016/s0001-2998(78)80014-2

[21] Swets JA (1988) Measuring the accuracy of diagnostic systems. Science 240: 1285–1293. 3287615 10.1126/science.3287615

[22] Giordano L, von Karsa L, Tomatis M, Majek O, de Wolf C, Lancucki L, et al (2012) Mammographic screening programmes in Europe: organization, coverage and participation. Journal of Medical Screening 19: 72–82. 10.1258/jms.2012.012085 22972813

[23] http://www.cancer.gov/types/breast/mammograms-fact-sheet.

[24] Sorkin RD, West R, Robinson DE (1998) Group performance depends on the majority rule. Psychological Science 9: 456–463.

[25] Hastie R, Kameda T (2005) The robust beauty of majority rules in group decisions. Psychological Review 112: 494 15783295 10.1037/0033-295X.112.2.494

[26] Wolf M, Kurvers RHJM, Ward AJW, Krause S, Krause J (2013) Accurate decisions in an uncertain world: collective cognition increases true positives while decreasing false positives. Proceedings of the Royal Society B: Biological Sciences 280: 20122777 10.1098/rspb.2012.2777 23407830 PMC3574371

[27] Carney PA, Bogart TA, Geller BM, Haneuse S, Kerlikowske K, Buist DSM, et al (2012) Association between time spent interpreting, level of confidence, and accuracy of screening mammography. American Journal of Roentgenology 198: 970–978. 10.2214/AJR.11.6988 22451568 PMC3654687

[28] Carney PA, Bogart A, Sickles EA, Smith R, Buist DSM, Kerlikowske K, et al (2013) Feasibility and acceptability of conducting a randomized clinical trial designed to improve interpretation of screening mammography. Academic Radiology 20: 1389–1398. 10.1016/j.acra.2013.08.017 24119351 PMC4152937

[29] Geller BM, Bogart A, Carney PA, Elmore JG, Monsees BS, Miglioretti DL (2012) Is Confidence of Mammographic Assessment a Good Predictor of Accuracy? American Journal of Roentgenology 199: W134–W141. 10.2214/AJR.11.7701 22733922 PMC3391746

[30] Onega T, Anderson ML, Miglioretti DL, Buist DSM, Geller B, Bogart A, et al (2013) Establishing a Gold Standard for Test Sets: Variation in Interpretive Agreement of Expert Mammographers. Academic Radiology 20: 731–739. 10.1016/j.acra.2013.01.012 23664400 PMC3741406

[31] Geller BM, Bogart A, Carney PA, Sickles EA, Smith R, Monsees B, et al (2014) Educational Interventions to Improve Screening Mammography Interpretation: A Randomized Controlled Trial. American Journal of Roentgenology 202: W586–W596. 10.2214/AJR.13.11147 24848854 PMC4276372

[32] Grofman B, Owen G, Feld SL (1983) Thirteen theorems in search of the truth. Theory and Decision 15: 261–278.

[33] Katsikopoulos KV, Martignon L (2006) Naive heuristics for paired comparisons: Some results on their relative accuracy. Journal of Mathematical Psychology 50: 488–494.

[34] Kurvers R, Wolf M, Krause J (2014) Humans use social information to adjust their quorum thresholds adaptively in a simulated predator detection experiment. Behavioral Ecology and Sociobiology 68: 449–456.

[35] Gromet M (2008) Comparison of computer-aided detection to double reading of screening mammograms: Review of 231,221 mammograms. American Journal of Roentgenology 190: 854–859. 10.2214/AJR.07.2812 18356428

[36] Dromain C, Boyer B, Ferre R, Canale S, Delaloge S, Balleyguier C (2013) Computed-aided diagnosis (CAD) in the detection of breast cancer. European Journal of Radiology 82: 417–423. 10.1016/j.ejrad.2012.03.005 22939365

[37] Dinnes J, Moss S, Melia J, Blanks R, Song F, Kleijnen J (2001) Effectiveness and cost-effectiveness of double reading of mammograms in breast cancer screening: findings of a systematic review. Breast 10: 455–463. 14965624 10.1054/brst.2001.0350

[38] Harvey SC, Geller B, Oppenheimer RG, Pinet M, Riddell L, Garra B (2003) Increase in cancer detection and recall rates with independent double interpretation of screening mammography. American Journal of Roentgenology 180: 1461–1467. 12704069 10.2214/ajr.180.5.1801461

[39] Helvie M (2007) Improving mammographic interpretation: double reading and computer-aided diagnosis. Radiologic Clinics of North America 45: 801–811. 17888770 10.1016/j.rcl.2007.06.004

[40] Shaw CM, Flanagan FL, Fenlon HM, McNicholas MM (2009) Consensus review of discordant findings maximizes cancer detection rate in double-reader screening mammography: Irish National Breast Screening Program experience. Radiology 250: 354–362. 10.1148/radiol.2502080224 19188311

[41] Kerr NL, Tindale RS (2004) Group performance and decision making. Annual Review of Psychology 55: 623–655. 14744229 10.1146/annurev.psych.55.090902.142009

[42] Bankier AA, Levine D, Halpern EF, Kressel HY (2010) Consensus interpretation in imaging research: is there a better way? Radiology 257: 14–17. 10.1148/radiol.10100252 20851935

