# Supplementary material for: Collective Intelligence Meets Medical Decision-Making: The Collective Outperforms the Best Radiologist
Source: PLoS One. 2015 Aug 12;10(8):e0134269. doi: 10.1371/journal.pone.0134269 (PMC4534443; doi:10.1371/journal.pone.0134269)
Supplement: S1 Text — (DOCX) [file pone.0134269.s001.docx]

**Supporting Information:**

**Collective intelligence meets medical decision-making: the collective outperforms the best radiologist**

Max Wolf^1^, Jens Krause^1,4^, Patricia A. Carney^2^, Andy Bogart^3^ & Ralf HJM Kurvers^1^

^1^ Leibniz Institute of Freshwater Ecology and Inland Fisheries. Müggelseedamm 310, 12587 Berlin, Germany

^2^ Departments of Family Medicine and Pubic Health & Preventive Medicine, Knight Cancer Institute, Oregon Health & Science University, 3181 S.W. Sam Jackson Park Road Portland, Oregon, USA

^3^ RAND Corporation, 1776 Main Street, Santa Monica, CA 90407-2138

^4^ Faculty of Life Sciences, Humboldt-University of Berlin, Germany

**Data collection**

Data used for this research were collected during the course of a mammographic test set study designed to evaluate an intervention designed to improve mammographic accuracy among community radiologists (*1, 2*). The study involved radiologists from U.S. breast cancer registries including Carolina Mammography Registry, San Francisco Mammography Registry, New Hampshire Mammography Network, New Mexico Mammography Project, Vermont Breast Cancer Surveillance System, and Group Health Cooperative in western Washington, all affiliated with the Breast Cancer Surveillance Consortium (BCSC). The data were assembled at the BCSC Statistical Coordinating Center (SCC) in Seattle and analysed at the Leibniz-Institute of Freshwater Ecology and Inland Fisheries (IGB) in Berlin, Germany. All registries as well as the SCC and IGB received institutional review board approval for either active or passive consenting processes or a waiver of consent to enroll participants, pool data and perform statistical analysis. All procedures are in accordance with the Health Insurance Portability and Accountability Act and all data were anonymized to protect the identities of women, radiologists and facilities.

Radiologists that interpreted mammograms at the facilities affiliated with the aforementioned registries between January 2005 and December 2006 were invited to participate in this study. Eligible non-BCSC radiologists from Oregon,Washington, North Carolina, San Francisco and New Mexico were also invited. In total, 403 radiologists were invited of which 101 completed all procedures and were included in this study.

182 unique mammography cases were used. These cases were randomly selected from screening examinations of women (40-69 years old) performed at the participating BCSC registries. Images from women that had a mastectomy and/or a known history of breast cancer were excluded. All the screening mammography examinations had a mammography study performed within the prior 11-30 months for comparison. Examinations with stray marks or other quality issues were excluded. Each case consisted of craniocaudal and mediolateral oblique views of each breast (four views per woman for each of the screening and comparison examinations). Cases in which cancer was diagnosed within 12 months after the mammograms were taken were selected as cancer cases. Cases that stayed cancer-free for a period of 2 years were selected as non-cancer cases. In total there were 51 cancer cases and 131 non-cancer cases.

Radiologists evaluated cases in two stages. For stage 1, four test sets were created, each containing 109 cases (*1*). Radiologists were randomly assigned to one of four test sets. For stage 2, one test set containing 110 cases was created. Some of these cases were already observed by part of the radiologists in part 1. To avoid using the same case twice per radiologist, we excluded all those cases from part 2 that were already viewed by that radiologist in part 1. This resulted in a total of 161 unique cases for radiologists belonging to test set 1 (*N*=25 radiologists) and 2 (*N*=30) and 173 unique cases for radiologists belonging to test set 3 (*N*=26) and 4 (*N*=20), resulting in 16,813 unique readings. In between both stages radiologists were randomly assigned to one of three intervention treatments. Since there were no strong treatment differences (*2*) we pooled the data from stage 1 and 2.

The selected cases were digitized (DiagnosticPro Advantage, Vidar Systems) to form the four test sets for stage 1 and the one test set for stage 2. These test sets were self-administered and distributed on a DVD. Radiologists viewed the test sets at a home or a work computer or at a laptop provided with the DVD. These laptops had a large screen and high-resolution graphics (≥ 1280 X 1024, ≥ 3GHz, 1 GB of RAM and a video-card with 128 MB allowing displaying full 32-bit colour). Radiologists saw two images at the same time (i.e., the left and right breast) and were able to quickly (≤ 1s) alter between paired images, to magnify a selected part of an image, and to select abnormalities by clicking on the screen. Radiologists were instructed to interpret the cases akin to clinical practice. The cases in each test set were shown in a random order. Each case was presented in a sequence including mediolateral and craniocaudal oblique views of both breasts simultaneously. This was followed by mediolateral and craniocaudal views of each breast paired with the analogous case from the previous examination to assess any changes in comparison with the previous mammogram.

During viewing each case, radiologists were asked whether or not a case should be recalled for further examination. The decision to recall constituted a positive test result in our analysis. We thus focus on the performance at the woman level.

**Data analysis**

*Majority rule*

In order to investigate the performance of the majority rule (Table 1, main text), and how this depends on group size, we repeatedly (see below) selected one of the four test sets at random, from which we then randomly drew *n* radiologists (range: 1 – 15, only using odd numbers to avoid the need for a tie-breaking rule). We selected radiologists within test sets since within each test set all radiologists viewed the same cases. We then classified each case as ‘recall’ or ‘no recall’ depending on which of these two options received most votes from the *n* radiologists. After classifying all cases in the test set in this way, we used the known cancer status of each case to calculate the average true and false positive rate and the overall accuracy (i.e., the fraction of correct decisions) achieved by groups of radiologists that employ the majority rule. For each group size *n*, this process was repeated 2,500 times.

*Quorum rule*

In order to investigate the performance of the quorum rule (Table 1, main text), and how this depends on group size, we repeatedly (see below) selected one of the four test sets at random, from which we then randomly selected *n* radiologists (range: 1 – 15, only using odd numbers). We then randomly assigned half of the mammograms from the selected test set to a training set and the remaining half to a validation set. The training set was used to determine the quorum threshold. We calculated the average true and false positive rate of the *n* radiologists in the training set and set the quorum threshold halfway between both values. We then investigated the performance of the quorum rule in the validation set. We stress that – as the training and the validation set contain different cases – we thus ensured to use different cases to (i) determine the quorum threshold and (ii) test its performance. Per case in the validation set we determined the fraction of *n* radiologists that classified the case as ‘recall’. If this fraction was higher than (or equal to) the quorum threshold, then the case was classified as ‘recall’, if not as ‘no recall’. After classifying all cases in the validation set in this way, we used the known cancer status of each case to calculate the average true and false positive rate and the overall accuracy obtained by the quorum rule. For each group size *n*, this process was repeated 2,500 times.

*Weighted quorum rule*

In order to investigate the performance of the weighted quorum rule (Table 1, main text), and how this depends on group size, we repeatedly (see below) selected one of the four test sets at random, from which we then randomly selected *n* radiologists (range: 1 – 15, only using odd numbers). We then randomly assigned half of the mammograms from the selected test set to a training set and the remaining half to a validation set. The training set was used to determine the quorum threshold (see above ‘Quorum rule’) and the weights. The weighted quorum rule prescribes that the vote of each radiologists is weighted according to its performance. In particular, according to a commonly employed rule for binary choice scenarios as investigated by us, if the performance of radiologist *i* is *p_i_,* then its vote has to be weighted by $w_{i}=\log(\frac{p_{i}}{1-p_{i}})$
(*3-5*). To determine these weights, for each radiologist, we used the training set to calculate its true positive rate and its true negative rate (i.e., 1 - false positive rate). We then calculated the performance value (*p_i_*) of each radiologist by averaging these two values and determined the associated weight *w_i_*. We note that this procedure implicitly gives equal weight to true and false positive rates, independent of the frequency of both conditions. Using these weights and the quorum threshold, we then determined the performance of the weighted quorum rule in the validation set. Per case in the validation set we summed the weights of radiologists that classified the case as ‘recall’. This weight was then divided by the total amount of weights of the *n* selected radiologists. If this fraction was higher than (or equal to) the quorum threshold, then the case was classified as ‘recall’, if not as ‘no recall’. After classifying all cases in the validation set in this way, we used the known cancer status of each case to calculate the average true and false positive rate and the overall accuracy obtained by the weighted quorum rule. For each group size *n*, this process was repeated 2,500 times.

*Best radiologist*

We compared the performance of the above CI-rules with the best radiologist within each group (see main text). In order to determine the performance of the best radiologist for a given group size *n,* we repeatedly (see below) selected one of the four test sets at random, from which we then randomly selected *n* radiologists. As above, we then randomly assigned half of the mammograms from the selected test set to a training set and the remaining half to a validation set. The training set was used to identify the best radiologist. In the training set, we calculated the true positive rate and the true negative rate of each radiologist and then selected the best performing radiologist, giving equal weight to the true positive rate and the true negative rate. We then calculated the performance (i.e., true and false positive rate and overall accuracy) of the best radiologist in the validation set. For each group size *n*, this process was repeated 2,500 times.

*Scenario 1-3 (Figure 3A-C)*

To maximize collective improvement in true positives, while keeping the false positive rate close to the average false positive rate in the data set (0.336), we repeatedly selected one of the four test sets at random, from which we then randomly selected *n* radiologists (range: 1 – 15, only using odd numbers). We then randomly assigned half of the mammograms from the selected test set to a training set and the remaining half to a validation set. We then used the training set to determine the quorum threshold that results in a false positive rate equal to the average individual false positive rate of 0.336. This threshold was then applied to the validation set. Similarly, to achieve maximum improvement in false positives, while keeping the true positive close to the average true positive rate in the date set (0.762), we used the training set to determine the quorum threshold that results in a true positive rate of 0.762 and investigated the performance of this quorum threshold in the validation set; to maximize accuracy, we determined the quorum threshold that maximized performance (i.e., the highest fraction of correct decisions) in the training set and investigated its performance in the validation set. For each of the three scenarios and each group size *n,* this process was repeated 2,500 times.

**References**

1. P. A. Carney *et al.*, Association Between Time Spent Interpreting, Level of Confidence, and Accuracy of Screening Mammography. *American Journal of Roentgenology* **198**, 970 (2012).

2. B. M. Geller *et al.*, Educational Interventions to Improve Screening Mammography Interpretation: A Randomized Controlled Trial. *American Journal of Roentgenology* **202**, W586 (2014).

3. B. Grofman, G. Owen, S. L. Feld, Thirteen theorems in search of the truth. *Theory and Decision* **15**, 261 (1983).

4. R. D. Sorkin, R. West, D. E. Robinson, Group Performance Depends on the Majority Rule. *Psychological Science* **9**, 456 (1998).

5. K. V. Katsikopoulos, L. Martignon, Naive heuristics for paired comparisons: Some results on their relative accuracy. *Journal of Mathematical Psychology* **50**, 488 (2006).
